# Absorption, Distribution, Metabolism, and Excretion of [^14^C]BS1801, a Selenium-Containing Drug Candidate, in Rats

**DOI:** 10.3390/molecules28248102

**Published:** 2023-12-15

**Authors:** Cheng Yang, Mingzhen Xue, Yifei He, Hanwei Yin, Chen Yang, Dafang Zhong, Huihui Zeng, Yuandong Zheng, Xingxing Diao

**Affiliations:** 1 School of Chinese Materia Medica, Nanjing University of Chinese Medicine, Nanjing 210023, China; yc2550983083@163.com; 2 Shanghai Institute of Materia Medica, Chinese Academy of Sciences, Shanghai 201210, China; s20-heyifei@simm.ac.cn (Y.H.); yangchen@simm.ac.cn (C.Y.); dfzhong@simm.ac.cn (D.Z.); 3 Jiangsu Key Laboratory for Functional Substances of Chinese Medicine, Nanjing University of Chinese Medicine, Nanjing 210023, China; xuemingzhen@foxmail.com; 4 Shanghai Yuanxi Pharmaceutical Technology Co., Ltd., Shanghai 201203, China; yinhanwei@yxmedicine.com

**Keywords:** [^14^C]BS1801, BS1801 (butaselen), tissue distribution, metabolism, mass balance, selenium

## Abstract

BS1801 is a selenium-containing drug candidate with potential for treating liver and lung fibrosis. To fully elucidate the biotransformation of BS1801 in animals and provide sufficient preclinical drug metabolism data for human mass balance study, the metabolism of BS1801 in rats was investigated. We used radiolabeling techniques to investigate the mass balance, tissue distribution, and metabolite identification of BS1801 in Sprague–Dawley/Long–Evans rats after a single oral dose of 100 mg/kg (100 μCi/kg) [^14^C]BS1801: 1. The mean recovery of radioactive substances in urine and feces was 93.39% within 168 h postdose, and feces were the main excretion route. 2. Additionally, less than 1.00% of the dose was recovered from either urine or bile. 3. BS1801-related components were widely distributed throughout the body. 4. Fifteen metabolites were identified in rat plasma, urine, feces, and bile, and BS1801 was detected only in feces. 5. BS1801-M484, the methylation product obtained via a N–Se bond reduction in BS1801, was the most abundant drug-related component in plasma. The main metabolic pathways of BS1801 were reduction, amide hydrolysis, oxidation, and methylation. Overall, BS1801 was distributed throughout the body, and excreted mainly as an intact BS1801 form through feces. No differences were observed between male and female rats in distribution, metabolism, and excretion of BS1801.

## 1. Introduction

The thioredoxin (Trx) system, which consists of Trx, thioredoxin reductase (TrxR), and nicotinamide adenine dinucleotide phosphate (NADPH), is a key antioxidant system that defends against oxidative stress by regulating the protein dithiol/disulfide balance through its disulfide reductase activity [1]. TrxR/Trx is involved in the development of various diseases, especially cancer [2,3]. In addition, the Trx system is essential for normal cellular function, so it is necessary to develop its inhibitors as a therapeutic strategy [4]. TrxR is a selenocysteine-containing enzyme responsible for maintaining redox homeostasis in cells and plays a key role in regulating diverse cellular redox signaling pathways [5]. However, few pharmaceutical companies have been actively involved in the development of TrxR enzyme inhibitors, and the inhibitors described in the literature and patents fall into the following main categories: metal complexes, Michael receptors, sulfur/selenium (Se)-containing compounds, and others [4]. Nevertheless, most of the disclosed inhibitors were developed based on the modification of the selenocysteine residue (the selenol group) of TrxR, which has an overall similar but relatively stronger response (nucleophilicity and metal-binding property) than that of its counterpart cysteine residue (the thiol group) [2,4,6]. Importantly, the concentration of thiols in cells is much higher than that of selenols, so the abundance of thiols compensates for their low reactivity; as a result, the inhibitor may undergo cross-reactivity with thiols [4]. Therefore, inhibitors of TrxR should be further developed.

In recent years, Se-containing compounds have attracted increasing attention. Se is an essential trace element for many biological functions and a component of selenoproteins [7]. It is generally believed that Se has multiple positive effects on human health [8,9]. A study summarizing more than 100 Se-containing compounds found that Se-containing compounds exhibit a variety of anticancer, antioxidant, and antifibrotic activities and that incorporating Se atoms into small molecules significantly increases their biological activity [6]. Different structures of Se derivatives have different efficacy [6,7]. Compared with the toxicity and adverse reactions of inorganic selenium compounds, organic selenium derivatives have attracted more attention, and many organic selenium compounds have been found to have strong anti-tumor activity [6,10,11]. Selenium-containing drugs are effective chemopreventive and chemotherapeutic agents for cancer [6,12]. Se is also ingested in the form of dietary supplements [13]. Additionally, the presence of Se affects the pharmacokinetics, metabolism, and detection of drugs. Se contains six stable isotopes with different abundances, which form characteristic peak clusters and facilitate the identification of compounds containing Se.

Ebselen (2-phenyl-1,2-benzisoselenazol-3(2H)-one), a nontoxic organoselenium compound discovered in the 1980s, was used to prevent and treat a variety of diseases because of its wide range of targets and ability to modulate a variety of biological processes [9,14,15]. The treatment of stroke in 1997 was not approved in Japan due to insufficient efficacy data [14]. However, researchers have not stopped exploring the compound, and it is currently in clinical stage III [16]. BBSKE (1,2-[bis(1,2-benzisoselenazolone-3 (2H)-ketone)]-ethane) is a heterocyclic organoselenium compound that has been reported to exhibit antitumor effects and is a novel antitumor agent [17]. Ebselen and BBSKE had a high binding rate with plasma proteins, resulting in their inability to perform pharmacokinetic studies by directly quantifying the parent drug. Several studies quantified the plasma metabolites (selenol derivatives) of ebselen or BBSKE via different sample pretreatment methods or the LC–MS method combined with other methods to assess their pharmacokinetics [18,19,20].

BS1801 (butaselen), a selenium-containing drug candidate, is a novel TrxR inhibitor developed by Shanghai Yuanxi Pharmaceutical Technology Co., Ltd. It is a structural analog of ebselen and BBSKE, all of which contain Se. Ebselen exerts its effects by mimicking glutathione peroxidase activity, whereas BS1801 and BBSKE exert their effects by inhibiting TrxR activity [21,22,23]. Although ebselen has been included in a number of clinical trials in the United States and Asia, there is no specific prospect of clinical application [24]. Studies on BBSKE have focused more on its anticancer activity, while the current drug candidate BS1801 has been proposed for the clinical treatment of fibrosis and is currently in phase Ⅰ clinical trials [17,25,26].

To our knowledge, there are only two studies on the metabolism and pharmacokinetics related to BS1801. In the first study, the covalent binding product of BS1801 and human serum albumin was described, as well as the mechanism of its reduction and methylation [27]. In another study, M2 (BS1801-M484) was identified as the major metabolite of BS1801 in human plasma via mass spectrometry response and UV absorption peak area, and the pharmacokinetics of M2 were evaluated [25]. However, the mass spectrometry and UV response do not accurately reflect the abundance of metabolites since they are susceptible to structural modifications of metabolites.

Among the available studies, there were no accurate data to investigate the proportion of metabolites in BS1801, so the safety of major metabolites cannot be evaluated. At the same time, there was a lack of tissue distribution and mass balance data for BS1801 in vivo. Since the concentration of the drug in the target organ is directly related to the efficacy and toxicity, tissue distribution must be investigated in animals to further advance the research progress of BS1801 for eventual marketing [28]. A mass balance study of radiolabeled BS1801 in humans is also needed. In the draft *Clinical Pharmacology Considerations for Human Radiolabeled Mass Balance Studies* published by the US FDA in May 2022, it was noted that the dose of human radioactive administration should be estimated based on animal research data [29]. Therefore, the radiolabeled mass balance study in animals must be performed before human-radiolabeled mass balance study.

**Figure 1:**
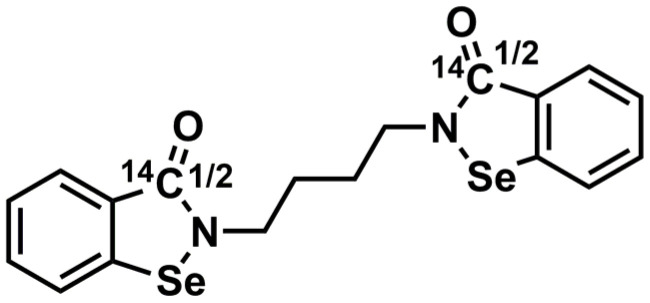
Chemical structure of [^14^C]BS1801.

In this study, the distribution, metabolism, and excretion of [^14^C]BS1801(Figure 1) in rats were studied. We evaluated the overall metabolism of BS1801 in rats, providing a strong basis for human mass balance study and promoting clinical research.

## 2. Results

### 2.1. Mass Balance

**Figure 2:**
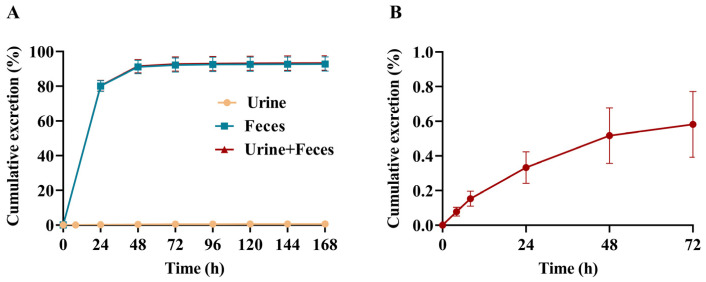
Mean cumulative excretion of total radioactivity (3 male and 3 female SD rats) in urine and feces (**A**) and bile (**B**) in SD rats after a single oral dose of 100 mg/kg (100 μCi/kg) [^14^C]BS1801.

The mean radioactive recovery in urine and feces was 93.39% in SD rats after a single oral administration from 0 to 168 h. Among them, feces was the main excretion route, and the cumulative excretion of radioactive substances accounted for 92.77% of the dose, while urine accounted for only 0.62%. The mean radioactive recovery in urine and feces within 0–48 h was 91.57% after administration. The cumulative radioactive recoveries of urine and feces are provided in Figure 2A (detailed data are provided in Supplemental Table S2).

The recovery of radioactivity in bile within 72 h after a single intragastric administration in SD rats undergoing BDC was 0.58% (Figure 2B and detailed data are provided in Supplemental Table S2). The drug excreted through the feces consisted mainly of the part absorbed via the bile and the unabsorbed part, and it was clear from the biliary excretion data that BS1801 in feces was predominantly unabsorbed.

### 2.2. Tissue Distribution

**Figure 3:**
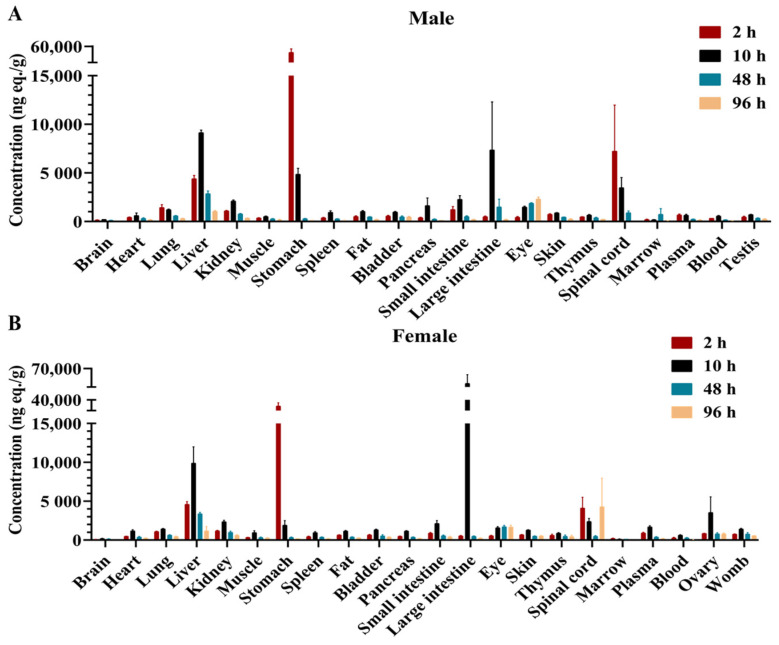
Tissue concentration–time distribution in male (**A**) and female (**B**) LE rats after a single oral dose of 100 mg/kg (100 μCi/kg) [^14^C]BS1801.

All tissues of twenty-four LE rats (3 male and 3 female at each time point) were collected at 2, 10, 48, and 96 h after intragastric administration. The drug-related substances in male rats were mainly concentrated in the liver, stomach, large intestine, and eyes (Figure 3A). The ratios of AUC_0–96h_ in these tissues to plasma AUC_0–96h_ were 11.80, 10.60, 7.48, and 5.35, respectively (Supplemental Table S3). In female rats, drug-related substances were mainly concentrated in the large intestine and liver, and the AUC_0–96h_ ratios of these tissues to the plasma AUC_0–96h_ were 16.82 and 6.59, respectively (Figure 3B and Supplemental Table S3). The ratios of other tissues AUC_0–96h_ to plasma AUC_0–96h_ were lower than 5.00 postdose in all rats (Supplemental Table S3).

In male LE rats, the *T*_max_ was 2 or 10 h for all tissues except the eyes and marrow. In female LE rats, the *T*_max_ was 2 or 10 h for all tissues except the eyes and spinal cord (Figure 3A,B).

The average ratio of blood AUC_0–96h_ to plasma AUC_0–96h_ was 0.73 in male rats postdose, while that of female rats was 0.45 (Supplemental Table S3), indicating that the drug-related substances preferred to not be distributed in blood cells.

### 2.3. Metabolite Identification

**Figure 4:**
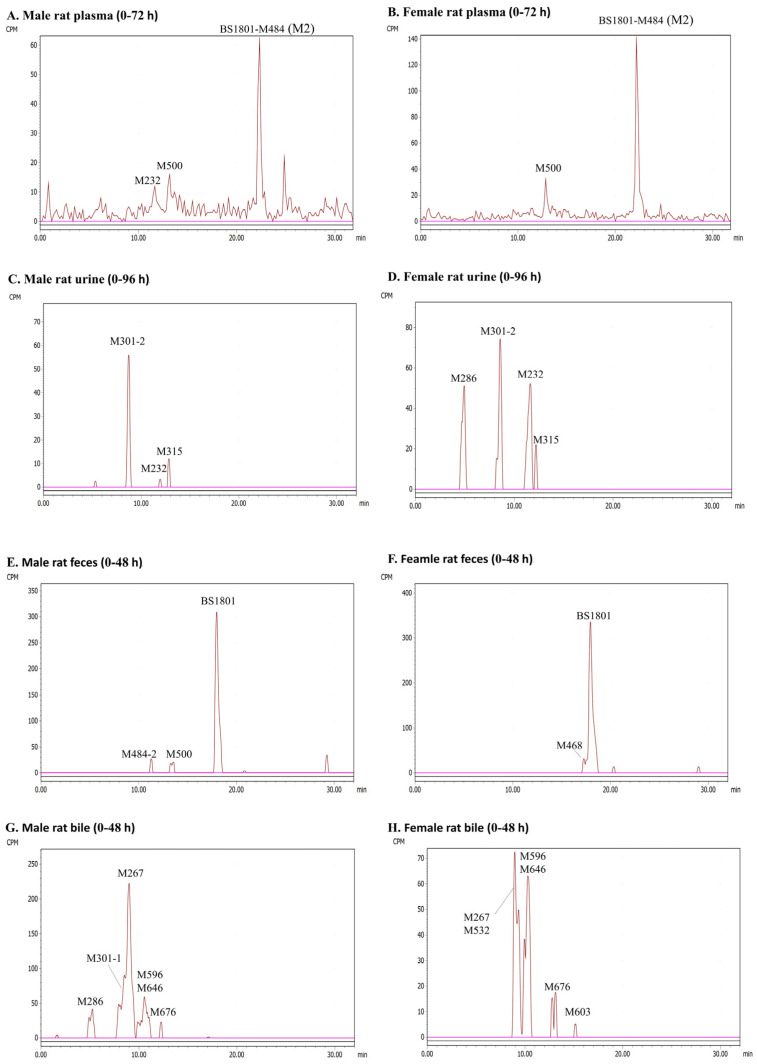
Representative radio-chromatograms of metabolites of BS1801 after a single oral dose of 100 mg/kg (100 μCi/kg) [^14^C]BS1801 in male and female SD rats. (**A**) Male rat plasma (0–72 h); (**B**) female rat plasma (0–72 h); (**C**) male rat urine (0–96 h); (**D**) female rat urine (0–96 h); (**E**) male rat feces (0–48 h); (**F**) female rat feces (0–48 h); (**G**) male rat bile (0–48 h); (**H**) female rat bile (0–48 h).

Figure 4 presents radio-chromatograms of pooled plasma (0–72 h), urine (0–96 h), feces (0–48 h), and bile (0–48 h) for male and female rats. A total of twelve metabolites in male rats and thirteen metabolites in female rats were identified after drug administration. Supplemental Table S4 summarizes the proposed metabolic pathways, elemental composition, determined *m*/*z* ([M + H]^+^), mass error (ppm), and characteristic fragment ions for BS1801 and its metabolites.

#### 2.3.1. HR-MS Analysis of BS1801 and M2 Reference Standards

**Figure 5:**
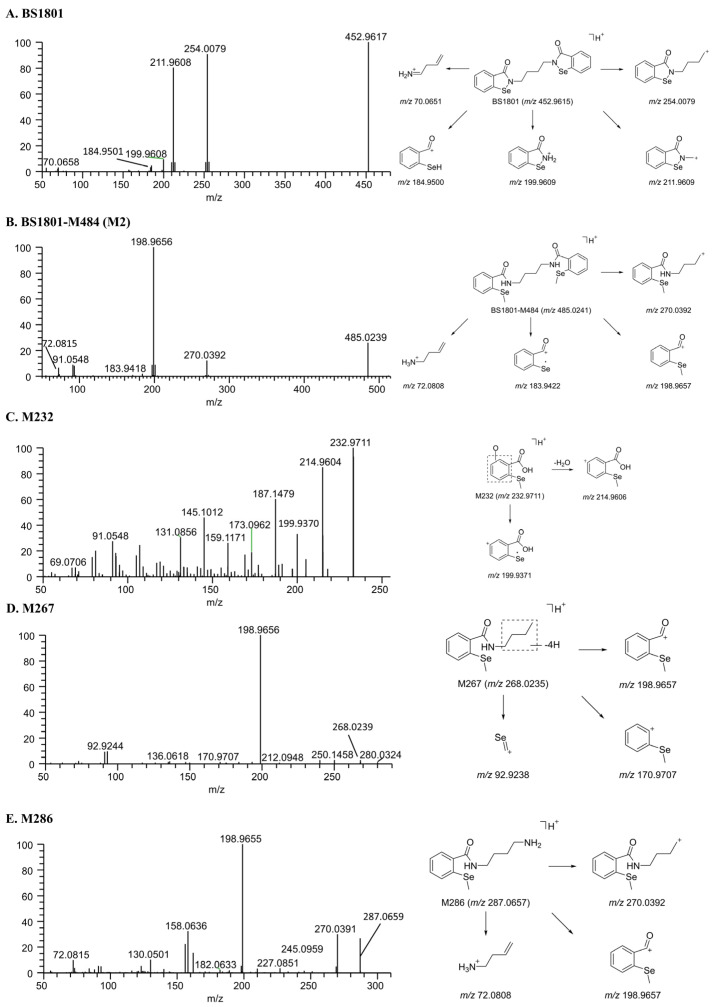
Mass spectra of metabolites and their proposed fragmentation profiles. (**A**) BS1801; (**B**) BS1801-M484 (M2); (**C**) M232; (**D**) M267; (**E**) M286.

BS1801. Under the experimental conditions of this study, BS1801 (C_18_H_16_N_2_O_2_Se_2_) eluted at 17.65 min, and the molecular ion ([M + H]^+^) obtained in the full-scan MS was *m*/*z* 452.9617. The major fragment ions of BS1801 were *m*/*z* 254.0079, 211.9608, 199.9608, and 184.9501. The fragment ions *m*/*z* 254.0079 and 199.9608 were mainly produced by C–N bond cleavage. The C–C bond breakage produced a fragment ion *m*/*z* 211.9608. The fragment ion *m*/*z* 184.9501 was produced by the amide bond breakage and N–Se bond breakage (Figure 5A).

M2. M2 was the product of BS1801 reduction and further methylation, and its retention time was 22.28 min. M2 yielded a molecular ion ([M + H]^+^) *m*/*z* 485.0242 and its major fragment ions *m*/*z* 270.0392, 198.9656, 183.9418, and 72.0815 in full-scan MS. The cleavage of the C–N bond produced a fragment ion *m*/*z* 270.0392, and the cleavage of the amide bond produced a fragment ion *m*/*z* 198.9656. Amide bond breakage and the loss of CH_3_· radical produced the fragment ion *m*/*z* 183.9418. The fragment ion *m*/*z* 72.0815 was produced by C–N bond breakage (Figure 5B).

#### 2.3.2. Plasma

BS1801 was not detected in AUC_0–72h_ pooled male rat plasma. Three metabolites were identified via HR-MS and radio-chromatographic peaks. Among them, M2 accounted for 65.41% of the total radioactivity and was the most abundant metabolite. M500 and M232 accounted for 20.07% and 11.59% of the total radioactivity, respectively. In female rats, BS1801 was also undetected in plasma. Only two metabolites, M2 and M500, were detected, accounting for 81.33% and 14.00% of the total plasma radioactivity, respectively (Figure 4, and Supplemental Table S5).

#### 2.3.3. Urine

The cumulative excretion of radioactive substances in urine was only 0.62% of the dose, as shown in the mass balance study. A total of three radioactive chromatographic peaks were identified in 0–96 h pooled male rat urine sample, and the parent drug BS1801 was not detected. M232, M301-2, and M315 accounted for 0.018%, 0.41%, and 0.068% of the dose, respectively. Four radioactive peaks were identified in 0–96 h pooled female rat urine sample, and BS1801 was not detected. M232, M286, M301-2, and M315 accounted for 0.23%, 0.20%, 0.21%, and 0.046% of the dose, respectively (Figure 4, and Supplemental Table S5).

#### 2.3.4. Feces

According to the mass balance study, feces was the main excretion route. The cumulative excretion of radioactive substances in male rats feces accounted for 92.32% of the dose, and BS1801 accounted for 76.97% of the dose. Two metabolites were identified by combining HR-MS and radio-chromatographic peaks. Among them, M500 was the major metabolite, accounting for 5.73% of the dose; M484-2 was the minor metabolite, accounting for 4.00% of the dose. The cumulative excretion of radioactive substances in the feces of female rats accounted for 93.22% of the dose, and BS1801 accounted for 81.89% of the dose. Only the metabolite M468 was identified by combining HR-MS and radio-chromatographic peaks, accounting for 7.77% of the dose (Figure 4, and Supplemental Table S5).

#### 2.3.5. Bile

The cumulative excretion of radioactive substances in bile accounted for only 0.58% of the dose. Six metabolites were identified using HR-MS and radio-chromatographic peaks in pooled 0–48 h male rat bile. Among them, M267 was the major metabolite, accounting for 0.46% of the dose. M301-1 was the second abundant metabolite, accounting for 0.22% of the dose. Co-eluting metabolites M596 and M646 accounted for 0.18% of the dose. The contents of M286 and M676 were less than 0.1% of the dose. Six metabolites were also identified in pooled female rat bile. Among them, M267 and M532 co-eluted; M596 and M646 also co-eluted. The contents of all metabolites in bile were less than 0.1% of the dose (Figure 4, and Supplemental Table S5).

#### 2.3.6. Description of Metabolites

BS1801 and M2. The structures of BS1801 and M2 were confirmed by comparison with the retention time and LC–MS/MS spectra of the reference standards.

M232. The metabolite of M232 (*m*/*z* 232.9713) was composed of C_8_H_8_O_3_Se, which was produced by the amide hydrolysis of M484-2. Its main fragment ions were *m*/*z* 214.9604 and 199.9370. The fragment ion *m*/*z* 214.9604 was 18.0109 Da (H_2_O) smaller than M232, which was obtained by the neutral loss of water from the precursor ion. The fragment ion *m*/*z* 199.9370 was produced by further losing CH_3_· radical from the precursor ion *m*/*z* 214.9604 (Figure 5C).

M267. The elemental composition of M267 (*m*/*z* 268.0237) was C_12_H_13_NOSe. The precise molecular weight of M267 was the same as that of M286 minus an NH_5_, corresponding to deamination and dehydrogenation. The main product ions were *m*/*z* 198.9656, 170.9707, and 92.9244. Among them, the product ion *m*/*z* 198.9656 was the same as the product ion *m*/*z* M286. This supports the inferred structure of M267 (Figure 5D).

M286. The elemental composition of M286 (*m*/*z* 287.0660) was C_12_H_18_N_2_OSe, which was produced by *N*-dealkylation and methylation of BS1801. The main fragment ions were *m*/*z* 270.0391, 198.9655, and 72.0815. Among them, the fragment ion *m*/*z* 270.0391 was produced by the neutral loss of NH_3_; fragment ion *m*/*z* 198.9655 was produced by amide bond cleavage (Figure 5E).

**Figure 6:**
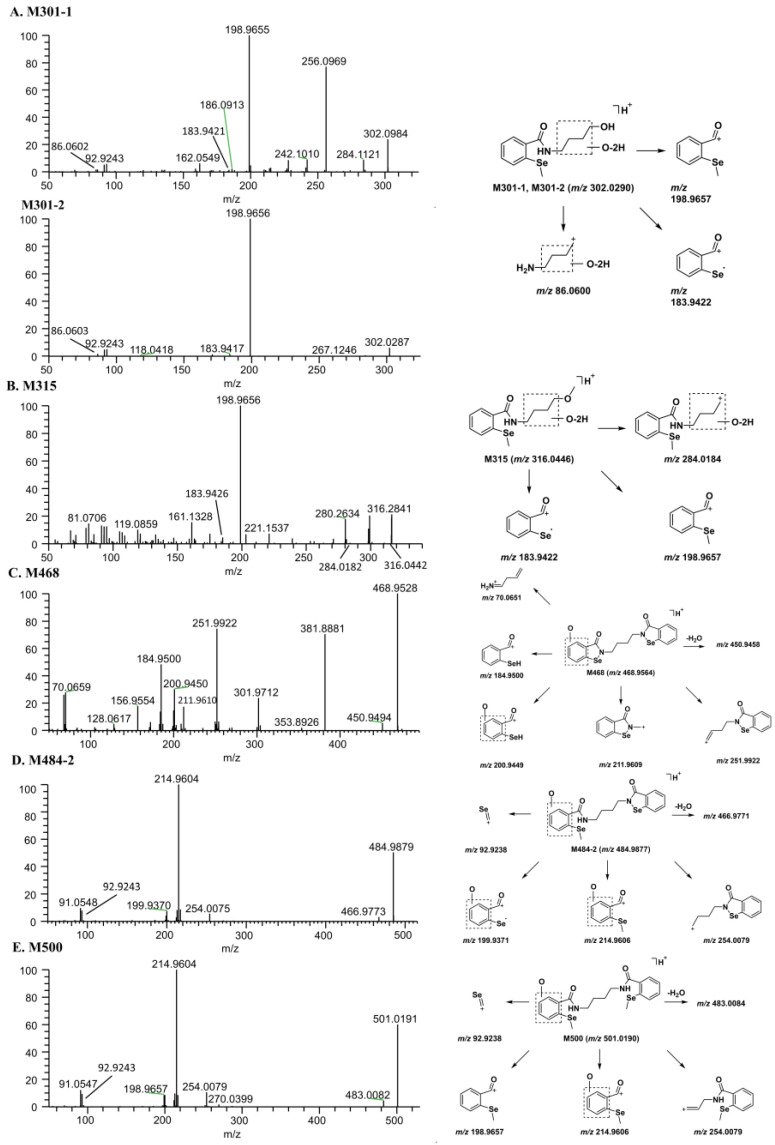
Mass spectra of metabolites and their proposed fragmentation profiles. (**A**) M301-1 and M301-2; (**B**) M315; (**C**) M468; (**D**) M484-2; (**E**) M500.

M301-1 and M301-2. M301-1 and M301-2 were produced by oxidative deamination and further oxidation of M286. The [M + H]^+^ of M301-1 and M301-2 (C_12_H_15_NO_3_Se) were *m*/*z* 302.0290, which was identical to the theoretical value. The main fragment ion *m*/*z* 198.9655 (the same as the fragment ions of M286) was produced by amide bond breakage, and the fragment ion *m*/*z* 183.9421 was produced by amide bond cleavage and losing CH_3_· radical. The fragment ion *m*/*z* 86.0602 supported the modification site of M301-1. The fragment ions of M301-2 were similar to those of M301-1 (Figure 6A).

M315. Metabolite M315 (*m*/*z* 316.0447) was composed of C_13_H_17_NO_3_Se, which was produced by M301 methylation. M315 produced the same fragment ions as M301 at *m*/*z* 198.9656 and 183.9426. The fragment ion *m*/*z* 284.0182 was the fragment of M315 after the neutral loss of methanol. These results indicated that M315 was a methylated product of M301 (Figure 6B).

M468. M468 (C_18_H_16_N_2_O_3_Se_2_), with [M + H]^+^ at *m*/*z* 468.9552, was 15.9945 Da (O) larger than that of BS1801, suggesting that M468 was produced by the mono-oxidation of BS1801. Its major product ions *m*/*z* 211.9610, 184.9500, and 70.0659 were identical to those of BS1801. The product ion *m*/*z* 450.9494 was obtained by the neutral loss of water from the precursor ion. The difference between the product ions *m*/*z* 200.9450 and 184.9500 was 15.9950 Da (O), so it was inferred that the oxidation site was on the benzene ring (Figure 6C).

M484-2. The [M + H]^+^ of M484-2, *m*/*z* 484.9880, was 16.0328 Da (CH_4_) larger than that of M468. Its elemental composition was C_19_H_20_N_2_O_3_Se_2_. Therefore, it was inferred that M484-2 was the product of Se methylation of M468. The fragment ion *m*/*z* 254.0075 was produced by C–N bond cleavage. Amide bond cleavage produced a fragment ion *m*/*z* 214.9604, and further losing CH_3_· radical produced a fragment ion *m*/*z* 199.9370. The fragment ion *m*/*z* 466.9773 was obtained from the neutral loss of water from M484-2 (Figure 6D).

M500. The [M + H]^+^ of M500, *m*/*z* 501.0192, was 15.9950 Da (O) larger than that of M2. According to the precise molecular weight, the molecular formula of M500 was C_20_H_24_N_2_O_3_Se_2_, indicating that it was derived from the mono-oxidation of M2. The main product ions of M500 were *m*/*z* 483.0082, 254.0079, 214.9604, 198.9657, and 92.9243. The product ion *m*/*z* 483.0082 was obtained by the neutral loss of water from the precursor ion. The product ion *m*/*z* 198.9657 was similar to the product ion of M2 and was 15.9947 Da (O) smaller than the product ion *m*/*z* 214.9604, indicating that the oxidation site was on the benzene ring (Figure 6E).

**Figure 7:**
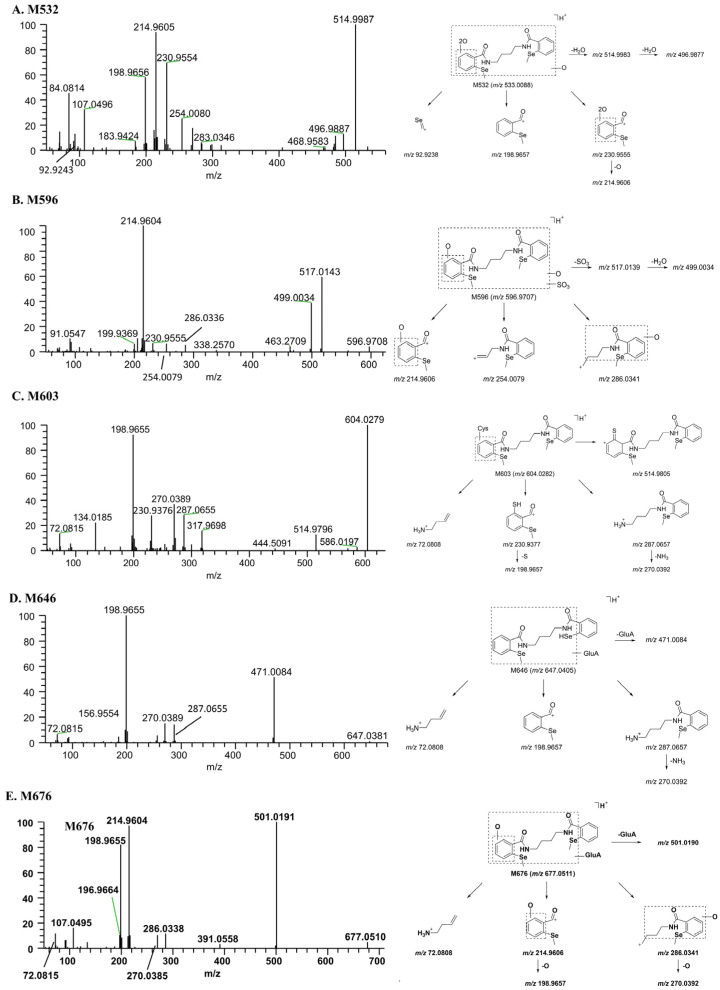
Mass spectra of metabolites and their proposed fragmentation profiles. (**A**) M532; (**B**) M596; (**C**) M603; (**D**) M646; (**E**) M676. Cys, cysteine conjugation; GluA, glucuronidation; SO_3_, sulfation.

M532. The [M + H]^+^ of M532 (C_20_H_24_N_2_O_5_Se_2_) was *m*/*z* 533.0091, which was produced by the di-oxidation of M500. The neutral loss of water from M532 produced fragment ion *m*/*z* 514.9987, and the further loss of water produced fragment ion *m*/*z* 496.9887. The breakage of the amide bond produced fragment ion *m*/*z* 230.9554, the further removal of one oxygen atom produced ion *m*/*z* 214.9605, and the removal of two oxygen atoms produced ion *m*/*z* 198.9656 (Figure 7A).

M596. The elemental composition of M596 (*m*/*z* 596.9709) was C_20_H_24_N_2_O_7_Se_2_S. M596 had one more SO_4_ than M500, indicating sulfation and oxidation. Its major product ions were *m*/*z* 517.0143 (the loss of SO_3_), 499.0034 (the loss of SO_3_ and water), 286.0336 (C–N bond breakage), 254.0079, and 214.9604. Among them, the product ions *m*/*z* 254.0079, and 214.9604 were identical to those of M500. However, the specific conjugation and oxidation sites were not determined (Figure 7B).

M603. The [M + H]^+^ of M603 (C_23_H_29_N_3_O_4_Se_2_S) was *m*/*z* 604.0291. Compared with M2, M603 was C_3_H_5_NO_2_S more than M2, corresponding to cysteine. The product ions *m*/*z* 270.0389, 198.9655, and 72.0815 of M603 were the same as those of M2. The product ion *m*/*z* 230.9376 was 31.9720 Da (S) more than the product ion *m*/*z* 198.9656 of M2; therefore, the binding site of M603 to cysteine was located on the benzene ring. The product ion *m*/*z* 514.9796 further supported the proposed structure of M603 (Figure 7C).

M646. The elemental composition of M646 (*m*/*z* 647.0406) was C_25_H_30_N_2_O_8_Se_2_. It was the product of BS1801 reduction, methylation, and glucuronidation. The fragment ion *m*/*z* 471.0084 was produced by the neutral loss of glucuronic acid. The breakage of the amide bond on one side yielded the product ions *m*/*z* 287.0655 and 198.9655. The neutral loss of NH_3_ (17.0266 Da) from the ion *m*/*z* 287.0655 generated the product ion *m*/*z* 270.0389 (Figure 7D).

M676. M676 (*m*/*z* 677.0506) had an elemental composition of C_26_H_32_N_2_O_9_Se_2_, which was C_6_H_8_O_6_ more than M500. After the neutral loss of a molecule of glucuronic acid (C_6_H_8_O_6_), M676 yielded product ion *m*/*z* 501.0191. When one side of the C–N or amide bond was broken, product ions *m*/*z* 286.0338 or 214.9604 were obtained; the further loss of an O atom yielded product ions *m*/*z* 270.0385 and 198.9655, respectively. The product ions *m*/*z* 214.9604 and 198.9655 were identical to M500 (Figure 7E).

**Figure 8:**
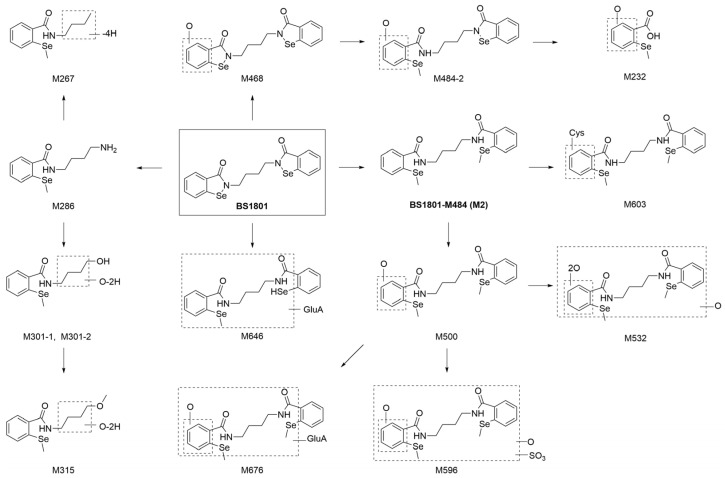
Metabolic pathway of BS1801 in rat. Cys, cysteine conjugation; GluA, glucuronidation; SO_3,_ sulfation. Bold means reference standards available.

The main metabolic pathways of BS1801 are shown in Figure 8.

## 3. Discussion

This study investigated the mass balance, tissue distribution, and metabolism of BS1801 in rats after a single oral dose of 100 mg/kg (100 μCi/kg) [^14^C]BS1801. BS1801 and its metabolites were identified in plasma, urine, feces, and bile via an accurate mass determination and diagnostic MS fragment ions.

In mass balance studies, the results measured with radiolabeling techniques are much more accurate than those measured with non-radiolabeled compounds [30]. In this study, 91.57% of the radioactivity was excreted within 48 h after oral administration of [^14^C]BS1801, indicating that [^14^C]BS1801 was rapidly excreted in rats. The mean cumulative radioactivity recovery in urine and feces was 93.39% within 168 h after administration, of which 92.77% was in feces, and only 0.62% was in urine, suggesting that feces was the main excretion route. The average cumulative radioactive recovery in bile was 0.58% of the dose. The radioactive recovery in either urine or bile was less than 1% of the dose; thus, we inferred that BS1801 was not well absorbed in rats (Supplemental Table S2). However, previous studies have shown that it has very good pharmacological effect, which may indicate that a small amount of BS1801 can exert a strong therapeutic effect [26]. Therefore, further research is needed to investigate whether BS1801 absorption may be improved by altering the dosage form, etc., which can minimize the administered dose. Drug-related substances were widely distributed after the intragastric administration of [^14^C]BS1801, and radioactivity was detected in all collected tissues. However, the overall distribution of radioactivity concentrations was low, which may be attributed to its low absorption. In male LE rats, high radioactivity concentration was detected mainly in the liver, stomach, large intestine, and eyes; in female LE rats, high radioactivity concentration was detected mainly in the large intestine and liver (Figure 3A,B). The relatively high concentration levels in the liver are favorable for the targeted treatment of liver fibrosis. The ratio of AUC_0–96h_ of brain to that of plasma was the lowest in all analyzed tissues, which may be due to the blood–brain barrier limiting the entry of BS1801 into the brain. The results of mass balance and tissue distribution of [^14^C]BS1801 in rats provide a basis for human dosimetry.

It is well known that high-resolution mass spectrometry is commonly used to quantitatively analyze compounds, but quantifying metabolite content is also crucial [31]. This study used high-resolution mass spectrometry combined with radio-chromatography to qualitatively and quantitatively analyze the metabolites associated with BS1801 simultaneously, which can provide the ratio of metabolites to dose. This will help us identify major metabolites that need further attention. This is the greatest advantage of radiolabeling technology, which can reflect the metabolite content and reveal the biotransformation pathway. Based on the high radioactive recovery of BS1801, a total of 15 metabolites were identified in plasma, urine, feces, and bile. The major metabolic pathways of BS1801 were reduction, amide hydrolysis, oxidation, and methylation, and the minor metabolic pathways were glucuronidation and sulfation (Figure 8). BS1801 was not detected in plasma, urine, and bile but was observed only in feces. Unchanged BS1801 was the major component in rat feces, accounting for more than 75.00% of the dose. M2, a product of methylation following reduction in the N–Se bond, was the major metabolite in plasma, accounting for 65.41% and 81.33% of plasma total radioactivity in male and female SD rats, respectively (Supplemental Table S5). This result is consistent with the findings obtained for unradiolabeled BS1801 in humans [25]. However, the metabolite of M2 was only present in plasma and was completely converted to other metabolites before excretion. In this study, M500 accounted for 20.07% and 14.00% of the plasma total radioactivity in male and female rats, respectively (Supplemental Table S5). This is inconsistent with a previous study in which the exposure of other metabolites was less than 5% of M2 [25]. This may result from several reasons, such as species differences or MS signal changes from metabolic modifications. Notably, it is likely that the MS response and UV peak area do not accurately reflect the metabolite content due to the influence of metabolic modifications. Additionally, we could not quantify each metabolite by using reference standards. In contrast, radiolabeling technology exhibits a unique advantage due to quantitative radioactivity unaffected by metabolite structure. M232 was a newly identified metabolite in the plasma of male rats, accounting for 11.59% of the total plasma radioactivity (Supplemental Table S5). Considering that M500 and M232 accounted for more than 10% of total radioactivity in plasma, we need to pay attention to their exposure in future human study. The advantages of the radiolabeling technique were demonstrated by comparing the results with those obtained by non-radiolabeled human plasma. Because the method can more accurately quantify the content of metabolites, some metabolites that are easily overlooked but need attention or safety assessment can be found.

BS1801 is further modified from the structure of ebselen and BBSKE. It has been reported that ebselen and BBSKE have high binding rates with albumin [19,32]. However, it is worth mentioning that when BS1801 plasma pooling was performed for protein precipitation, the recoveries of radioactivity in the supernatants of male and female rats were 105.85% and 91.23%, respectively, which demonstrated that the bottom-layer precipitation was almost free of BS1801 and its related metabolites. It has been reported that BS1801 can bind to human serum albumin rapidly and thoroughly, but the covalent binding is unstable; thus, the compound can be reduced and eventually metabolized to BS1801-M484 (M2) [25]. This explains why the bottom-layer protein precipitates in our study contain almost no radioactive substances. According to the free drug hypothesis, after compounds bind to plasma proteins, only free compounds are used for hepatic uptake through active transport or passive diffusion, and only free drugs can reach target tissues to interact with the target [29,33,34]. The properties of BS1801 covalently bound to albumin and eventually metabolized to the free product M2 are more conducive to drug efficacy. The pharmacokinetic/pharmacodynamic modeling (PK/PD modeling) of BS1801 needs to be further explored.

## 4. Materials and Methods

### 4.1. Chemicals and Reagents

BS1801 (99.5% content) and the related reference standard M2 (97.7% content) were provided by Shanghai Yuanxi Pharmaceutical Technology Co., Ltd. (Shanghai, China). [^14^C]BS1801 (Figure 1, 99.71% content) was provided by Wuxi Beta Co., Ltd. (Wuxi, China). The liquid scintillation solution was purchased from Zhejiang Dingxin (Shaoxing, China). The alkaline liquid scintillation cocktail was obtained from RDC^TM^ (Hilladale, NJ, USA). Acetonitrile and methanol of high-performance liquid chromatography (HPLC) grade were purchased from Sigma (St. Louis, MO, USA). Formic acid was purchased from Rhawn Chemical Reagent Co., Ltd. (Shanghai, China). Ammonium acetate was purchased from Sinopharm (Shanghai, China). Ultrapure water was obtained from a Milli-Q integral water purification system (Millipore, Molsheim, France). All other reagents used were HPLC grade or analytical grade.

### 4.2. Animals

Sprague–Dawley (SD, *Rattus norvegicus*) rats (6–8 weeks, 200–300 g) and Long–Evans (LE, *Rattus norvegicus*) rats (6–8 weeks, 160–220 g) were purchased from Zhejiang Charles River Laboratory Animals Co., Ltd. (Jiaxing, China). The animal use license number of Shanghai Institute of Materia Medica, Chinese Academy of Sciences is SYXK (Hu) 2020-0042. The ethics committee approval number for this study is 2022-07-DXX-06, and it was valid from July 2022 to July 2023. Healthy eligible rats were housed in cages containing toys for 2–5 days in a 12 h day/night constant-temperature laboratory with free access to food and water after receipt in cages with no more than 5 rats per cage. The rats were fasted for 12 h prior to drug administration. The Animal Ethics Committee of the Shanghai Institute of Materia Medica, Chinese Academy of Science (Shanghai, China) approved the study protocol. The study design is shown in Supplementary Table S1.

### 4.3. Dosing Solutions and Oral Administration

BS1801 and [^14^C]BS1801 were prepared in 0.5% sodium carboxymethyl cellulose solution at a target concentration of 10 mg/mL (10 μCi/mL). The radioactivity of [^14^C]BS1801 was measured using a liquid scintillation counter (LSC) (Hidex, Turku, Finland) before and after administration, and a single dose of 10 mL/kg was administered orally.

### 4.4. Plasma Samples for Metabolite Identification

Three SD rats of each sex were used to obtain plasma samples. Approximately 200 μL of blood was collected from the jugular vein of six SD rats in a centrifuge tube containing heparin sodium before and 0.5, 2, 4, 6, 10, 12, 24, 48, 72, and 96 h after administration, followed by centrifugation (3500× *g*, 5 min, 4 °C), to obtain plasma. All samples were stored at −20 °C for analysis.

### 4.5. Mass Balance

Three male and three female SD rats were housed individually in metabolic cages. Urine samples were collected from each rat at predose and 0–8, 8–24, 24–48, 48–72, 72–96, 96–120, 120–144, and 144–168 h postdose. When each urine sample was collected, the metabolic cage was flushed with a small amount of water for 0–72 h and methanol/water (50:50, *v*:*v*) for 72–168 h, and the flushing solution was added to the urine sample for the corresponding period. Fecal samples were also collected at predose and 0–24, 24–48, 48–72, 72–96, 96–120, 120–144, and 144–168 h postdose. Urine and fecal samples were weighed and recorded during each collection period. All samples were stored at −20 °C for testing.

Bile duct cannulation (BDC) was performed in 3 male and 3 female SD rats before drug administration. After the operation, the wounds were wiped with iodophor for 2 days, and painkillers were administered appropriately. The state of the rats was observed daily, and the drug was administered only when no abnormality was observed within 2 days. Bile was collected before and 0–4, 4–8, 8–24, 24–48, and 48–72 h after dosing. The weights of the bile samples were recorded, and the samples were stored at −20 °C until testing.

### 4.6. Tissue Distribution

Twenty-four LE rats (12 of each sex) were used for tissue distribution studies. Three male and three female rats were sacrificed through abdominal aortic bleeding at 2, 10, 48, and 96 h after administration. Approximately 1/5 of the blood was stored frozen at −20 °C, and the remaining blood was centrifuged (3500× *g*, 5 min, 4 °C) to obtain plasma. The brain, heart, lung, liver, stomach wall, spleen, kidney, muscle, pigmented fat, ovary, womb, testis, bladder wall, pancreas, small intestine wall, large intestine wall, eye, pigmented skin, thymus, spinal cord, and marrow were also collected. The tissues were washed with saline, dried, and stored at −20 °C until testing.

### 4.7. Sample Preparation for Radioactivity Analysis

#### 4.7.1. Urine, Bile, and Plasma Samples

Samples of urine (100 μL), bile (30 μL), and plasma (100 μL from the tissue distribution study) were weighed and added to 15 mL scintillation solution. Among them, urine and bile samples were duplicated, and plasma samples were in singlicate. The radioactivity was measured directly via LSC.

#### 4.7.2. Feces, Blood, and Tissue Samples

Approximately three times the weight of methanol/water (50:50, *v*:*v*) was added to each fecal sample and homogenized with a homogenizer (SKGZ, Shanghai Biheng, Shanghai, China). Approximately 0.2~0.3 g of the fecal homogenate was weighed in a combustion boat, and dried overnight. Blood samples (100 μL) and tissue samples (approximately 0.1 g) were weighed into burning boats. After the samples were completely dried, they were combusted using a Biological Sample Oxidizer (OTCS-11/1, Shanghai Yixing, Shanghai, China). The ^14^CO_2_ generated was captured with 15 mL alkaline scintillation solution; then, the radioactivity was measured using LSC.

### 4.8. Metabolite Radio-Profiling and Characterization

#### 4.8.1. Sample Preparation

The plasma samples (predose and 0.5, 2, 4, 6, 10, 12, 24, 48, and 72 h postdose) were pooled according to the AUC-pooling principle [35,36]. Then, 3 mL of methanol/water (50:50, *v*:*v*) was slowly added to the pooled plasma (1 mL), followed by vortexing for 1 min and sonication for 10 min twice. Then, the upper extract and the lower precipitate were separated by centrifugation (5000 rpm, 10 min). The supernatant was transferred to another new centrifuge tube. Then, 1 mL of water was added to the precipitate, vortexed for 1 min, and sonicated for 10 min. Then, 2 mL of methanol/acetonitrile (50:50, *v*:*v*) was added to the mixture, two vortexes were performed for 1 min, and the mixture was ultrasonicated for 10 min, followed by centrifugation (5000 rpm, 10 min). The two-times supernatant extracts were combined and dried under a nitrogen stream and finally redissolved with acetonitrile/water (20:80, *v*:*v*). The recoveries of extraction and reconstitution processes were measured via LSC.

Urine samples were pooled based on equal percentage according to the volume of urine from 0–8, 8–24, 24–48, 48–72, and 72–96 h, and the corresponding volumes of urine samples were mixed with the same gender. The pooled urine was vortexed and centrifuged (5000 rpm, 10 min). After centrifugation, the supernatant was dried under a nitrogen stream and then redissolved in acetonitrile/water (20:80, *v*:*v*). The bile samples at 0–4, 4–8, 8–24, and 24–48 h were pooled according to the same process as for urine.

The feces samples were pooled based on equal percentage according to the feces weight at 0–24 and 24–48 h for the same sex. The pooled fecal homogenate (1 g) was extracted by using the same method as for the plasma. The supernatant was then evaporated under a nitrogen stream and redissolved in 300 μL acetonitrile/water (20:80, *v*:*v*).

#### 4.8.2. UHPLC-β-RAM/UV/QE MS Analysis

An ACQUITY UPLC BEH Pheny column (100 × 2.1 mm, 1.7 μm) with a Phenomenex KrudKatcher Ultra HPLC In-Line Filter guard column (2 µm, Depth Filter × 0.004 in ID) was used to perform metabolite radio-profiling and characterization on the Vanquish UHPLC system, which was combined with a QExactive Plus mass spectrometer (Thermo, Waltham, MA, USA) containing an electrospray ionization (ESI) source. The column temperature was 40 °C, and the flow rate was 0.45 mL/min. The mobile phases contained 5 mM ammonium acetate with 0.1% formic acid in water (A) and acetonitrile (B). The gradient elution procedure was as follows: 0 min, 5% B; 2 min, 10% B; 27 min, 40% B; 28 min, 95% B; 31 min, 95% B; 32 min, 5% B; and 35 min, 5% B. The eluent was monitored using a photodiode array detection with a full range of wavelengths. The mass spectrometer was operated in positive (+ESI) mode with a *m*/*z* 100–1500 Da scanning range. The optimized MS parameters were as follows: sheath gas, 35 L/min; auxiliary gas, 10 L/min; capillary temperature, 320 °C; and spray voltage, 3.8 kV. Data acquisition was performed using Xcalibur 4.1 software (Thermo, Waltham, MA, USA), and data analysis was accomplished using Compound Discoverer software 3.3 SP2 (Thermo, Waltham, MA, USA).

The extracted plasma, urine, feces, and bile samples were separated via UHPLC and detected using an online/offline radioactivity detector to obtain the corresponding radioactive metabolite profiles. Due to low radioactivity of plasma, the UHPLC eluent was collected using a UHPLC fraction collector (Thermo, Waltham, MA, USA) over time (10 s/well) on Deepwell LumaPlateTM 96-well plates. After evaporation via a vacuum centrifuge concentrator, the radioactivity was determined with an offline Sense Beta detector (1 min/well, Hidex, Turku, Finland). For the urine, feces, and bile samples, the UHPLC eluent was analyzed using an online radioactivity detector β-RAM. Radioactive metabolite profiling was integrated to obtain the radio-chromatogram with Laura 6.0.5.101 software (Lablogic, Sheffield, UK). The percentage of each metabolite in urine, feces, and bile (%dose) and the ratio of metabolites in the plasma (% area under the curve, %AUC) were calculated.

## 5. Conclusions

In brief, this study investigated the distribution, metabolism, and excretion of [^14^C]BS1801 in rats. The mean recovery of radioactive substances in urine and feces was 93.39% in SD rats within 168 h postdose, and feces was the main excretion route with the cumulative excretion of radioactive substances accounting for 92.77% of the dose. Additionally, less than 1.00% of the dose was recovered from either urine or bile. The main excretion period of [^14^C]BS1801 after administration was within 48 h, during which the mean radioactive dose recovery in urine and feces reached 91.57%. Drug-related substances were mainly distributed in the liver, stomach, large intestine, and eyes, and high concentrations in the liver facilitate targeted liver fibrosis therapy. A total of fifteen metabolites of BS1801 were detected in rats, and the main metabolic pathways were reduction, amide hydrolysis, oxidation, and methylation. Consistent with most thiol metabolites, the Se site was susceptible to methylation. BS1801-M484, the methylation product obtained by reduction in the N–Se bond in BS1801, was the most abundant drug-related component, and its radioactivity accounted for 65.41% and 81.33% of the pooled AUC_0–72h_ plasma in male and female rats, respectively. In conclusion, these data are expected to provide a strong basis for further clinical studies.

## Data Availability

The research data used to support the findings of this study are included in the article.

## Supplementary Materials

The following are available online at https://www.mdpi.com/article/10.3390/molecules28248102/s1, Table S1: Study design. Table S2: Mean cumulative dose recovery in urine, feces, urine + feces, and bile following a single oral administration of [^14^C]BS1801 at the dose of 100 mg/kg (100 μCi/kg) to SD rats. Table S3: AUC_0–96h_ of radioactivity in tissues and plasma of rats, and their ratio to plasma following a single oral administration of [^14^C]BS1801 at the dose of 100 mg/kg (100 μCi/kg). Table S4: Information of BS1801and its metabolites detected in rat plasma, urine, feces, and bile by using UHPLC-Q Exactive Plus MS. Table S5: The distribution of radioactivity from BS1801 and its major metabolites in rat plasma, urine, feces, and bile and the percent of dose in urine and feces (%).
