# Supplementary material for: Absorption, Distribution, Metabolism, and Excretion of [14C]BS1801, a Selenium-Containing Drug Candidate, in Rats
Source: Molecules. 2023 Dec 15;28(24):8102. doi: 10.3390/molecules28248102 (PMC10745422; doi:10.3390/molecules28248102)
Supplement: Supplementary file 1 [file molecules-28-08102-s001.zip › molecules-2761907-supplementary.pdf]

Supplemental Table S1

Study design.

Group	Matrix collection	administration	Number and Gender	Dosage			Time period or point in time for sample collection (h)
				mg/kg	μCi/kg	mL/kg	
Mass balance	Urine and feces	PO	3 male and 3 female rats	100	100	10	0, 0-8, 8-24 (feces, 0-24), 24-48, 48-72, 72-96, 96-120, 120-144, 144-168
	Bile	PO	3 male and 3 female rats	100	100	10	0, 0-4, 4-8, 8-24, 24-48, 48-72
Tissue distribution	Brain, heart, lung, liver, stomach wall, spleen, kidney, muscle, colored fat, ovary, womb, testis, bladder wall, pancreas, small intestine wall, large intestine wall, eye, colored skin, thymus, spinal cord, marrow, plasma and blood	PO	12 male and 12 female rats	100	100	10	2, 10, 48 and 96
Metabolite identification	Plasma	PO	3 male and 3 female rats	100	100	10	0.5, 2, 4, 6, 10, 12, 24, 48, 72 and 96
	Urine	Urine, fecal and bile samples of metabolite identification study were obtained from mass balance study.					0, 0-8, 8-24, 24-48, 48-72 and 72-96
	Feces						0, 0-24, 24-48
	Bile						0, 0-4, 4-8, 8-24 and 24-48

Supplemental Table S2

Mean cumulative dose recovery in urine, feces, urine + feces, and bile following a single oral administration of [^{14}C]BS1801 at the dose of 100 mg/kg (100 μCi/kg) to SD rats.

Time (h)	Urine (%)			Feces (%)			Urine + Feces (%)			Bile (%)		
	Male (n=3)	Female (n=3)	Mean	Male (n=3)	Female (n=3)	Mean	Male (n=3)	Female (n=3)	Mean	Male (n=3)	Female (n=3)	Mean
0	0.00	0.00	0.00	0.00	0.00	0.00	0.00	0.00	0.00	0.00	0.00	0.00
0 - 4	-	-	-	-	-	-	-	-	-	0.12	0.034	0.077
0 - 8	0.028	0.051	0.039	-	-	-	0.028	0.051	0.039	0.23	0.073	0.15
0 - 24	0.19	0.26	0.22	83.68	76.51	80.10	83.87	76.77	80.32	0.50	0.16	0.33
0 - 48	0.36	0.46	0.41	90.65	91.67	91.16	91.02	92.13	91.57	0.85	0.19	0.52
0 - 72	0.45	0.58	0.52	91.65	92.84	92.24	92.10	93.42	92.76	0.98	0.19	0.58
0 - 96	0.49	0.67	0.58	91.98	93.03	92.50	92.47	93.69	93.08	-	-	-
0 - 120	0.51	0.70	0.61	92.13	93.11	92.62	92.64	93.82	93.23	-	-	-
0 - 144	0.51	0.72	0.61	92.23	93.16	92.70	92.74	93.87	93.31	-	-	-
0 - 168	0.51	0.73	0.62	92.32	93.22	92.77	92.83	93.95	93.39	-	-	-

-, no sample

Supplemental Table S3

$AUC_{0\text{-}96h}$ of radioactivity in tissues and plasma of rats, and their ratio to plasma following a single oral administration of [^{14}C]BS1801 at the dose of 100 mg/kg (100 μCi/kg).

Tissue	Male (n=3)							Female (n=3)						
	concentration (ng eq./g)				$AUC_{0\text{-}96h}$ (ng eq.×h/g)	R	P	concentration (ng eq./g)				$AUC_{0\text{-}96h}$ （ng eq.×h/g)	R	P
	2 h	10 h	48 h	96 h				2 h	10 h	48 h	96 h			
Brain	160.94	216.40	133.81	0.00	11535.82	0.36	0.00%	68.68	197.20	135.42	0.00	10702.14	0.17	0.00%
Heart	439.95	629.04	328.01	174.46	34959.16	1.08	27.73%	489.96	1187.56	390.52	253.84	52648.10	0.82	21.37%
Lung	1446.42	1223.73	586.70	312.38	68102.97	2.11	21.60%	1100.89	1443.91	638.94	450.63	77003.61	1.19	31.21%
Liver	4412.92	9147.10	2872.73	1042.46	380994.58	11.80	11.40%	4607.42	9895.21	3393.12	1188.76	425061.50	6.59	12.01%
Kidney	1096.95	2106.07	787.40	340.64	95957.87	2.97	16.17%	1177.14	2357.26	1006.17	613.60	118094.03	1.83	26.03%
Muscle	371.83	521.44	280.55	151.45	29550.72	0.91	29.04%	334.00	956.79	312.71	270.20	43607.69	0.68	28.24%
Stomach	43159.64	4868.26	289.62	96.10	342528.31	10.60	0.22%	25902.89	1929.12	338.68	199.96	193246.66	3.00	0.77%
Spleen	375.64	947.74	281.98	79.25	37703.20	1.17	8.36%	459.40	963.82	368.19	182.97	44688.48	0.69	18.98%
Fat	520.91	1038.13	485.15	208.75	52352.90	1.62	20.11%	659.49	1158.63	404.15	261.36	53597.12	0.83	22.56%
Bladder	565.26	989.51	493.52	457.33	57782.40	1.79	46.22%	676.64	1336.37	550.28	374.07	66759.38	1.04	27.99%
Pancreas	392.43	1650.33	252.62	128.20	53859.46	1.67	7.77%	488.14	1169.65	369.22	213.47	50342.31	0.78	18.25%
Small intestine	1235.98	2294.33	516.38	199.51	85942.05	2.66	8.70%	885.21	2145.95	556.20	391.31	87091.12	1.35	18.24%
Large intestine	505.96	7359.03	1512.19	198.78	241582.38	7.48	2.70%	534.35	45862.73	479.21	253.54	1084205.80	16.82	0.55%
Eye	436.21	1498.60	1877.18	2314.26	172909.82	5.35	100.00%	559.15	1581.33	1724.34	1674.09	153491.05	2.38	97.09%
Skin	740.91	886.34	454.92	245.58	49545.97	1.53	27.71%	686.62	1289.98	512.19	524.41	67712.47	1.05	40.65%
Thymus	480.75	666.53	383.18	226.15	39638.48	1.23	33.93%	624.36	883.64	489.33	441.56	55083.86	0.85	49.97%
Spinal cord	7240.81	3491.23	911.95	0.00	155716.12	4.82	0.00%	4123.23	2397.65	478.43	4294.41	199400.34	3.09	100.00%
Marrow	197.43	182.94	739.41	95.75	39287.33	1.22	12.95%	208.61	121.03	117.17	7.09	9035.22	0.14	3.40%
Plasma	676.02	659.11	241.95	139.90	32301.05	1.00	20.70%	907.77	1698.81	386.69	176.30	64470.34	1.00	10.38%
Blood	336.15	565.24	165.18	70.76	23482.33	0.73	12.52%	285.94	635.22	282.53	25.14	28791.98	0.45	3.96%

Tissue	Male (n=3)							Female (n=3)						
	concentration (ng eq./g)				$AUC_{0\text{-}96h}$ (ng eq.×h/g)	R	P	concentration (ng eq./g)				$AUC_{0\text{-}96h}$ （ng eq.×h/g)	R	P
	2 h	10 h	48 h	96 h				2 h	10 h	48 h	96 h			
Testis	476.61	706.30	360.34	250.29	40129.68	1.24	35.44%	-	-	-	-	-	-	-
Ovary	-	-	-	-	-	-	-	846.37	3554.21	790.70	803.17	139254.86	2.16	22.60%
Womb	-	-	-	-	-	-	-	761.65	1430.80	779.07	554.56	83525.91	1.30	38.76%

$AUC_{0\text{-}96h}$, Area under the tissue concentration-time curve from time 0 to 96 h; R, The ratio of $AUC_{0\text{-}96h}$ in tissue to that in plasma; P, The ratio of concentration at 96 h to C_{max} in tissue; -, no sample.

Supplemental Table S4

Information of BS1801and its metabolites detected in rat plasma, urine, feces, and bile by using UHPLC-Q Exactive Plus MS.

ID	Pathway	Formula	$[M+H]^+$ *m/z* (determined)	Mass error (ppm)	Fragment ions
BS1801	Parent drug	$C_{18}H_{16}N_2O_2Se_2$	452.9617	-1.8	254.0078, 211.9608, 199.9608, 184.9501, 70.0658
BS1801-M484 (M2)	2[2H]+2[CH$_2$]	$C_{20}H_{24}N_2O_2Se_2$	485.0242	0.2	270.0394, 198.9656, 183.9416, 72.0815
M232	M484-2+amide hydrolysis	$C_8H_8O_3Se$	232.9713	0.9	214.9604, 199.9370
M267	M286-NH$_3$+[-2H]	$C_{12}H_{13}NOSe$	268.0237	0.7	198.9656, 170.9707, 92.9244
M286	2[2H]+2[CH$_2$]+amide hydrolysis	$C_{12}H_{18}N_2OSe$	287.0660	1.0	270.0391, 198.9655, 72.0815
M301-1	M286+oxidative deamination+[O]+[-2H]	$C_{12}H_{15}NO_3Se$	302.0290	0.0	198.9655, 183.9421, 86.0602
M301-2	M286+oxidative deamination+[O]+[-2H]	$C_{12}H_{15}NO_3Se$	302.0290	0.0	198.9656, 183.9417, 86.0603
M315	M301+[CH$_2$]	$C_{13}H_{17}NO_3Se$	316.0447	0.3	284.0182, 198.9656, 183.9426
M468	[O]	$C_{18}H_{16}N_2O_3S_{e2}$	468.9552	-2.6	450.9494, 251.9922, 211.9610, 200.9450, 184.9500, 70.0659
M484-2	M468+[2H]+[CH$_2$]	$C_{19}H_{20}N_2O_3Se_2$	484.9880	0.6	466.9773, 254.0075, 214.9604, 199.9370, 92.9243
M500	BS1801-M484+[O]	$C_{20}H_{24}N_2O_3Se_2$	501.0192	0.4	483.0082, 254.0079, 214.9604, 198.9657, 92.9243
M532	M500+2[O]	$C_{20}H_{24}N_2O_5Se_2$	533.0091	0.6	514.9987, 496.9887, 230.9554, 214.9605, 198.9656, 92.9243
M596	M500+[O]+[SO$_3$]	$C_{20}H_{24}N_2O_7Se_2S$	596.9709	0.3	517.0143, 499.0034, 286.0336, 254.0079, 214.9604
M603	BS1801-M484+[Cys]	$C_{23}H_{29}N_3O_4Se_2S$	604.0291	1.5	514.9796, 287.0655, 270.0389, 230.9376, 198.9655, 72.0815
M646	2[2H]+[CH$_2$]+[GluA]	$C_{25}H_{30}N_2O_8Se_2$	647.0406	0.2	471.0084, 287.0655, 270.0389, 198.9655, 72.0815
M676	M500+[GluA]	$C_{26}H_{32}N_2O_9Se_2$	677.0506	-0.7	501.0191, 286.0338, 270.0385, 214.9604, 198.9655, 72.0815

The HR-MS and HR-MS^2 acquisition tests were performed using a Vanquish UHPLC system coupled with a Q Exactive Plus mass spectrometer equipped with an ESI source. All possible metabolites were input into the inclusion list, and the MS/MS data were acquired when the target *m/z* ions were detected.

[CH$_2$], methylation; [Cys], cysteine conjugation; [GluA], glucuronidation; [2H], hydrogenation; [O], oxidation; [SO$_3$], sulfation.

Supplemental Table S5

The distribution of radioactivity from BS1801 and its major metabolites in rat plasma, urine, feces, and bile and the percent of dose in urine and feces (%).

ID	Retention time (min)		Plasma (%AUC)		Urine (%Dose)		Feces (%Dose)		Bile (%Dose)		Urine + Feces (%Dose)	
	M	F	M	F	M: 0.51% of dose	F: 0.73% of dose	M: 92.32% of dose	F: 93.22% of dose	M: 0.98% of dose	F: 0.19% of dose	M: 92.83% of dose	F: 93.95% of dose
BS1801	18.10	17.88	-	-	-	-	76.97	81.89	-	-	76.97	81.89
BS1801-M484 (M2)	22.33	22.17	65.41	81.33	-	-	-	-	-	-	-	-
M286	5.22	4.90	-	-	-	0.20	-	-	0.084	-	-	0.20
M301-1	8.38	-	-	-	-	-	-	-	0.22	-	-	-
M301-2	8.78	8.57	-	-	0.41	0.21	-	-	-	-	0.41	0.21
M267	8.93	8.90[a]	-	-	-	-	-	-	0.46	0.088[a]	-	-
M532	-		-	-	-	-	-	-	-		-	-
M596	10.78[a]	10.35[a]	-	-	-	-	-	-	0.18[a]	0.081[a]	-	-
M646			-	-	-	-	-	-			-	-
M484-2	11.28	-	-	-	-	-	4.00	-	-	-	4.00	-
M232	11.67-11.93	11.60	11.59	-	0.018	0.23	-	-	-	-	0.018	0.23
M676	12.25	12.98	-	-	-	-	-	-	0.022	0.018	-	-
M315	12.85	12.18	-	-	0.068	0.046	-	-	-	-	0.068	0.046
M500	13.17-13.42	12.83	20.07	14.00	-	-	5.73	-	-	-	5.73	-
M603	-	15.17	-	-	-	-	-	-	-	0.0028	-	-
M468	-	17.57	-	-	-	-	-	7.77	-	-	-	7.77

M, male SD rat; F, female SD rat; %AUC is derived from the $AUC_{0\text{-}72h}$ pooled plasma; %Dose is derived from 0-168 h of urine and feces excretion data; -, no obvious radioactive peak in radio-chromatogram; [a], co-eluted.
